# Preclinical amyloid pathology is associated with anxiety but not depression in cognitively normal older adults: Evidence for differential neuropsychiatric pathways

**DOI:** 10.1016/j.tjpad.2026.100497

**Published:** 2026-01-30

**Authors:** Jonathan Vogelgsang, Clara Beck, Regan Patrick, Ipsit Vahia, Sara Weisenbach

**Affiliations:** a McLean Hospital, Mass General Brigham Department of Psychiatry, Harvard Medical School, MA, USA; b University Medical Center, Department of Psychiatry, Goettingen, Germany; c Dermatologikum Hamburg, Germany

**Keywords:** Amyloid-beta, Anxiety, Depression, Alzheimer’s disease, Stress, A4 study

## Abstract

**Introduction:**

Neuropsychiatric symptoms may represent early Alzheimer's disease (AD) manifestations, but their relationship with amyloid pathology in cognitively unimpaired individuals remains unclear.

**Methods:**

We analyzed 4,492 cognitively unimpaired adults (aged 65-85) from the Anti-Amyloid Treatment in Asymptomatic Alzheimer's Study. Participants completed amyloid-PET imaging and assessments of anxiety, depression, memory complaints, and AD concerns.

**Results:**

1,231 participants (27.4%) were amyloid-positive. While anxiety and depression scores remained within normal ranges, amyloid-positive participants reported higher memory complaints (p = 0.008) and AD concerns (p < 0.001) before status disclosure. Regression analyses showed amyloid burden associated with anxiety (β = 0.04, standardized, p = 0.003) but not depression (p = 0.68). Mediation analyses revealed anxiety was directly associated with amyloid, while depression was mediated through subjective cognitive concerns.

**Discussion:**

Preclinical amyloid pathology directly associates with subclinical anxiety but only indirectly with depression through subjective stress, suggesting distinct neuropsychiatric pathways in early AD that could inform detection strategies.

## Highlights

• Preclinical amyloid pathology directly associates with anxiety in cognitively normal elderly
• Depression links to amyloid only indirectly through subjective cognitive concerns
• Anxiety may represent an early neurobiological marker of AD pathology
• Subjective AD concerns mediate the relationship between amyloid and depression

## 1 Background

Alzheimer’s disease (AD) is a progressive neurodegenerative disorder and the most common cause of dementia worldwide, affecting millions of individuals and placing a significant burden on patients, caregivers, and healthcare systems [[1], [2], [3]]. Pathologically, AD is characterized by the accumulation of extracellular β-amyloid (Aβ) plaques and intracellular neurofibrillary tangles composed of hyperphosphorylated tau protein [[4], [5], [6], [7]]. The “amyloid hypothesis” has guided much of the research to date, positing that Aβ deposition triggers a cascade culminating in tau pathology and neurodegeneration [8,9]. However, new therapeutic approaches, including monoclonal antibodies targeting Aβ, have produced both excitement and debate regarding the precise role of amyloid in disease progression [10].

Historically, the clinical emphasis in AD has been on hallmark cognitive impairments, such as memory loss and executive dysfunction. Increasing evidence, however, points to neuropsychiatric symptoms (NPS) as critical components of the disease continuum, often emerging prior to overt cognitive deficits [[11], [12], [13], [14], [15], [16], [17], [18]]. These symptoms – encompassing anxiety, depression, and apathy, among others – have been associated with faster disease progression and diminished quality of life [14,15]. In the preclinical stages of AD, such symptoms may remain subtle yet reflect an underlying pathophysiological process, presenting challenges for early detection [12,13].

A related construct, known as subjective cognitive decline (SCD), refers to self-perceived deterioration of cognitive ability in the absence of objective deficits [19]. Individuals with SCD often report heightened stress, anxiety, and depressive symptoms, as well as an elevated likelihood of progressing to mild cognitive impairment (MCI) and AD [17,20,21]. Mounting evidence suggests that perceived memory complaints in cognitively normal individuals may relate to underlying amyloid pathology [22,23]. Moreover, chronic stress and elevated cortisol levels can further exacerbate amyloid accumulation and neuroinflammation, highlighting a possible bidirectional relationship between stress and early AD pathology [24,25].

While previous studies have examined either amyloid relationships with mood symptoms or with subjective cognitive concerns separately, few have investigated the interrelationships between all three elements in cognitively unimpaired individuals. This complex interplay may represent an important opportunity to identify psychological markers of preclinical AD before cognitive symptoms manifest.

Within this context, the Anti-Amyloid Treatment in Asymptomatic Alzheimer’s (A4) Study provides a unique opportunity to investigate older adults who are cognitively unimpaired yet harbor elevated amyloid levels. By examining the interplay between neuropsychiatric symptoms, subjective stress, and amyloid burden, we can elucidate whether amyloid accumulation influences early psychological responses despite normal cognitive performance. Additionally, determining how disclosure of amyloid status might affect anxiety, depression, or stress is increasingly relevant as biomarker-based diagnoses enter clinical and research paradigms [26,27].

Accordingly, our primary objectives were to: (1) compare anxiety, depression, and subjective stress between amyloid-positive and amyloid-negative cognitively unimpaired individuals; and (2) evaluate whether subjective stress mediates any observed relationships between amyloid burden and neuropsychiatric outcomes. We hypothesized that those with elevated amyloid burden would report higher subjective stress and potentially more pronounced anxiety or depressive symptoms – indicating that NPS may manifest at a preclinical stage of AD. By examining these psychological correlates of preclinical amyloid pathology, the present study aims to inform our understanding of the neuropsychiatric manifestations that may accompany early disease processes in individuals who remain cognitively intact.

## 2 Methods

### 2.1 Study design and data source

This cross-sectional analysis uses data from the Anti-Amyloid Treatment in Asymptomatic Alzheimer’s (A4) Study, which began in 2014 to investigate cognitively unimpaired older adults (65–85 years) with elevated β-amyloid (Aβ) on positron emission tomography (PET). De-identified A4 data were accessed via the Laboratory of Neuro Imaging (LONI) Image and Data Archive (IDA) under an approved data-use agreement.

We focused on Visit 1 (screening) and Visit 2 (PET analysis). At Visit 1, participants underwent cognitive and psychiatric evaluations; at Visit 2, they completed an amyloid-PET scan. Data from subsequent visits were not considered for this manuscript (e.g., Visit 3: disclosure of amyoid status).

### 2.2 Study population

Participants were included if they:

1. Were 65–85 years old;
2. Had no clinically significant cognitive impairment, defined by a Mini-Mental State Examination (MMSE) score of 25–30 and a Clinical Dementia Rating (CDR) of 0;
3. Completed amyloid-PET imaging at Visit 2.

The A4 protocol excluded individuals with clinically significant depression defined as GDS scores > 5 at screening, as well as those with a history of major depression or bipolar disorder within the past 2 years. Similarly, while formal anxiety disorders were not systematically assessed, individuals with severe psychiatric conditions that could interfere with study participation were excluded. Thus, all participants in this sub-analysis had GDS scores ≤ 5 at baseline, though this does not preclude lifetime history of mood disorders beyond the 2-year window. While this cutoff rules out formal depressive disorders, GDS still captures subclinical variations in mood. 4,492 participants underwent amyloid-PET and psychometric evaluation and were included in this study.

### 2.3 Baseline assessments (Visit 1)

• Mini-Mental State Examination (MMSE) [28]: A 30-point screening of global cognition; scores ≥ 25 are considered normal. Additional cognitive measures included the Wechsler Memory Scale-Revised (WMS-R) Logical Memory II subtest, with scores ranging from 6-18 required for inclusion. However, no measure of premorbid cognitive functioning was available.
• Clinical Dementia Rating (CDR) [29]: Rates dementia severity; a score of 0 indicates no dementia.
• Logical Memory-II Test [30]: Assesses immediate and delayed recall of standardized stories.
• Geriatric Depression Scale (GDS, 15-item) [31]: Fifteen yes/no items (range 0–15), with higher totals reflecting more depressive symptoms.
• State-Trait Anxiety Inventory (STAI, 6-item short form) [32]: Six statements rated on a 4-point Likert scale (1 = “Not at all” to 4 = “Very much so”); total scores range 6–24. For the purpose of this set of analyses, we considered only state anxiety.
• Memory Complaint Questionnaire (MCQ) [33]: Six items on everyday memory lapses, each rated on a 5-point scale (1 = “Never” to 5 = “Always”).
• Concerns about Alzheimer’s Disease (CAD) ([34] adapted in A4): Six statements rated on a 5-point scale (“Strongly Disagree” to “Strongly Agree”), capturing general worry about AD.

### 2.4 Amyloid PET imaging (Visit 2)

Participants with MMSE ≥ 25 and CDR = 0 proceeded to amyloid-PET imaging with 18F-Florbetapir. Uptake in six cortical regions was compared to a cerebellar reference region to generate a composite standardized uptake value ratio (SUVR). A composite SUVR ≥ 1.15 defined amyloid-positive (Aβ+), whereas < 1.15 defined amyloid-negative (Aβ−). The screening process allowed up to 90 days between Visit 1 (mood assessment) and Visit 3, with Visit 2 (PET imaging) occurring between these visits.

### 2.5 Data management and statistical analysis

All analyses were performed in R version 4.4.3 [35] . The following packages were used: dplyr [36], car [37], psych [38], lavaan [39], brunnermunzel [40]. For continuous variables, we assessed distributional properties prior to analysis. Given our large sample size (N = 4,492), we applied the Welch's t-test for group comparisons, which does not assume equal variances and is robust to non-normality in large samples per the Central Limit Theorem. For ordinal Likert-scale items from the Concerns about Alzheimer's Disease questionnaire, we employed the Brunner-Munzel test, a non-parametric alternative appropriate for ordinal data that does not assume normal distribution.

### 2.6 Group comparisons

To compare Aβ+ and Aβ− participants on continuous scales (e.g., GDS, STAI), an independent-samples t-test was used when normality assumptions were met. Categorical variables were compared using chi-square tests, with an alpha level of 0.05 (two-tailed).

### 2.7 Regression models

We employed linear regression (via lm()) to examine the association between amyloid burden (Aβ+ vs. Aβ− or continuous SUVR) and neuropsychiatric outcomes (GDS, STAI, MCQ, CAD). Each model controlled for age, sex, marital status, and education - covariates known to influence psychiatric and cognitive functioning in older adults. Effect sizes (unstandardized β, unless otherwise specified) and 95% confidence intervals were reported for interpretability.

### 2.8 Mediation analysis

To determine whether subjective stress mediates the relationship between amyloid burden and neuropsychiatric symptoms, we conducted mediation analyses using structural equation modeling with the lavaan package in R. We specified two potential mediators: subjective memory complaints (MCQ) and concerns about developing Alzheimer's disease (CAD). The mediation model examined whether amyloid burden (SUVR) influenced anxiety (STAI) and depressive symptoms (GDS) directly or indirectly through these subjective stress measures, while controlling for age, gender, marital status, and education.

For each outcome (STAI and GDS), we estimated: (1) the direct effect of amyloid on the outcome, (2) the indirect effects through each mediator, and (3) the total effect (sum of direct and indirect effects). This approach allowed us to quantify how much of amyloid's influence on neuropsychiatric symptoms operates through subjective stress versus direct neurobiological pathways. Significance of indirect effects was determined using standard errors estimated from the model, with significance level set at α = 0.05. We note that while mediation analysis with cross-sectional data can identify statistical indirect associations, it cannot establish causal directionality. Our mediation models should be interpreted as describing patterns of association rather than causal mechanisms.

### 2.9 Ethical considerations

All participants provided written informed consent, and the A4 protocol adhered to the Declaration of Helsinki. As these data were de-identified and accessed via a data-use agreement, no additional local IRB review was necessary. Further details regarding the A4 trial design and rationale are described in [41,42].

## 3 Results

### 3.1 Sample characteristics

A total of 4,492 cognitively unimpaired participants underwent amyloid-PET imaging and neuropsychological assessments as part of this study. Based on standardized uptake value ratio (SUVR) thresholds of 1.15, participants were classified as amyloid-positive (Aβ+) or amyloid-negative (Aβ−). Demographic factors, including age, sex, education level, and marital status, were examined to ensure comparability between groups. As summarized in Table 1, no significant differences were found between amyloid-positive and amyloid-negative participants in these baseline characteristics, suggesting that any observed effects in neuropsychiatric outcomes are likely attributable to amyloid burden rather than demographic variability. It is important to note that mean scores for both anxiety (STAI: 9.94 ± 3.11) and depression (GDS: 1.03 ± 1.47) were well within normal ranges, with GDS scores particularly low (floor effects), limiting clinical interpretation of these findings.

**Table 1:** Baseline characteristics of study participants by amyloid status.

	Total (N = 4492)	Amyloid Negative (n = 3261)	Amyloid Positive (n = 1231)	P Value
Amyloid load, composite SUVR, mean (SD)	1.09 (0.19)	1.00 (0.07)	1.35 (0.17)	<.001
Age, y, mean (SD)	71.29 (4.67)	70.89 (4.50)	72.36 (4.95)	<.001
Sex, No. (%)				.94
Male	1824 (40.6)	1323 (40.6)	501 (40.7)	
Female	2668 (59.4)	1938 (59.4)	730 (59.3)	
Marital status, No. (%)				.81
Married	3170 (70.6)	2301 (70.6)	869 (70.6)	
Widowed	427 (9.5)	306 (9.4)	121 (9.8)	
Divorced	629 (14.0)	455 (14.0)	174 (14.1)	
Single	183 (4.1)	134 (4.1)	49 (4.0)	
Unknown	83 (1.8)	65 (2.0)	18 (1.5)	
Education, y, mean (SD)	16.58 (2.83)	16.59 (2.85)	16.57 (2.79)	.84
GDS score, mean (SD)ᵃ	1.03 (1.47)	1.03 (1.50)	1.05 (1.30)	.68
STAI score, mean (SD)ᵇ	9.94 (3.11)	9.90 (3.12)	10.07 (3.09)	.10

Abbreviations: GDS, Geriatric Depression Scale; STAI, State-Trait Anxiety Inventory; SUVR, standardized uptake value ratio. ᵃ GDS scores range from 0 to 15, with higher scores indicating more depressive symptoms. ᵇ STAI scores range from 6 to 24, with higher scores indicating greater anxiety.

### 3.2 Subjective memory complaints, anxiety, and concerns about Alzheimer’s disease

A key objective of this study was to examine whether subjective cognitive complaints, anxiety, and concerns about AD differed between amyloid-positive and amyloid-negative participants. We first assessed self-reported memory complaints using the Memory Complaint Questionnaire (MCQ). Amyloid-positive individuals reported slightly higher subjective memory complaints compared to their amyloid-negative counterparts (p = 0.008). Although statistically significant, the absolute difference in MCQ scores between amyloid-positive and amyloid-negative groups was relatively small. This suggests that while subjective cognitive concerns may be detectable at a group level in preclinical AD, the magnitude of self-reported concerns at this stage remains subtle and might be difficult to detect clinically in individual patients without sensitive instruments. However, this finding aligns with prior literature suggesting that individuals with preclinical amyloid pathology may experience subtle, yet noticeable, changes in memory function even before overt cognitive impairment becomes apparent [22,43] . These self-perceived memory complaints could be driven by heightened self-monitoring or anxiety about cognitive decline, rather than objective cognitive deficits.

We then examined anxiety symptoms using the State-Trait Anxiety Inventory (STAI). Contrary to expectations, levels of anxiety were similar between amyloid-positive and amyloid-negative individuals (p = 0.10), indicating that amyloid burden alone does not necessarily translate to heightened anxiety in cognitively unimpaired individuals. Similarly, depressive symptoms, as assessed by the Geriatric Depression Scale (GDS), did not significantly differ between amyloid-positive and amyloid-negative participants (p = 0.68), suggesting that amyloid positivity does not directly impact mood symptoms at this stage of cognitive functioning.

### 3.3 Concerns and risk perceptions in amyloid-positive individuals

**Fig. 1:**
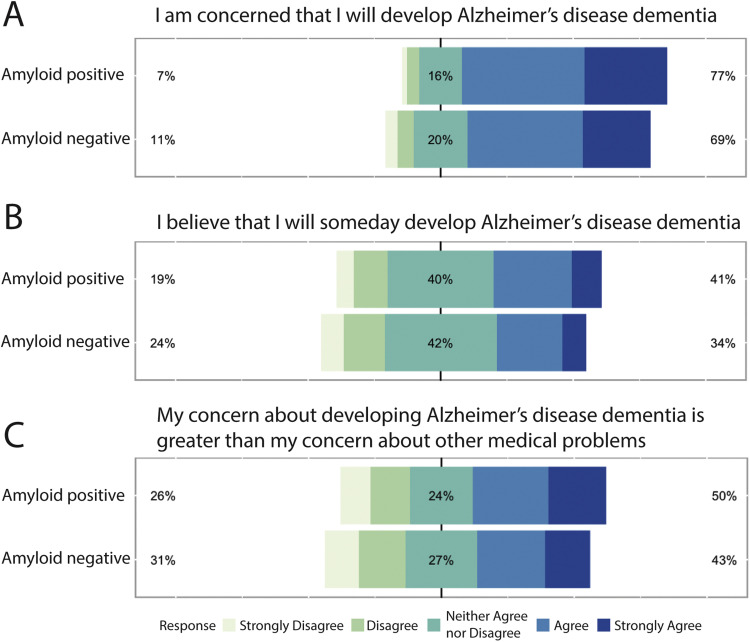
**Distribution of Concerns About Alzheimer's Disease by Amyloid Status.** Likert-scale responses to three items from the Concerns about Alzheimer's Disease (CAD) questionnaire comparing amyloid-positive (n = 1,231) and amyloid-negative (n = 3,261) participants. (A) "I am concerned that I will develop Alzheimer's disease dementia," (B) "I believe that I will someday develop Alzheimer's disease dementia," and (C) "My concern about developing Alzheimer's disease dementia is greater than my concern about other medical problems." Percentages on the left indicate participants who selected "Strongly Disagree" or "Disagree," while percentages on the right indicate those who selected "Agree" or "Strongly Agree." The center portion represents "Neither Agree nor Disagree" responses. All comparisons between amyloid-positive and amyloid-negative groups were statistically significant (Brunner-Munzel test; all P < .001).

Fig. 1 illustrates how participants rated their subjective experiences on a Likert scale. Specifically, amyloid-positive individuals tended to rate their concerns about memory problems and Alzheimer’s disease higher than amyloid-negative individuals (Fig. 1a). Fig. 1b demonstrates that these individuals also expressed heightened concern about developing Alzheimer’s disease. This suggests that amyloid burden is associated with increased worry about cognitive decline, even in the absence of objective impairment. While these concerns were more prominent in the amyloid-positive group, individual variability was present, highlighting the complex interaction between perceived risk, cognitive appraisal, and emotional responses.

Additionally, Fig. 1c illustrates that amyloid-positive individuals ranked AD as a more worrisome condition compared to other medical issues. Importantly, these three statements were analyzed using the Brunner-Munzel test due to their five-point ordinal response format:

1. “I am concerned that I will develop Alzheimer’s disease dementia.”
  • Aβ+ participants scored significantly higher than Aβ− (p = 3.82e-09).
2. “I believe that I will someday develop Alzheimer’s disease dementia.”
  • Aβ+ participants showed a greater belief in future AD onset (p = 3.18e-06).
3. “My concern about developing Alzheimer’s disease dementia is greater than my concern about other medical problems.”
  • Aβ+ participants rated AD as more worrisome than other health issues (p = 6.69e-06).

Even before receiving their amyloid status disclosure, these individuals reported significantly greater concerns about developing AD compared to amyloid-negative participants (see Fig. 1a–c). Amyloid-positive participants reported greater concerns about developing AD prior to status disclosure. While the mechanism underlying this association is unclear, possibilities include subtle subjective experiences not captured by standard cognitive testing, heightened self-monitoring in individuals who may have noticed minor changes, or shared underlying factors (e.g., personality traits, health awareness) that influence both concern levels and factors associated with amyloid accumulation. Our cross-sectional data cannot distinguish among these possibilities.

Having established group differences in subjective experiences, we next examined the quantitative relationship between amyloid burden and neuropsychiatric outcomes through regression analyses, allowing us to assess whether effects persisted after controlling for potential confounders.

### 3.4 Association between amyloid load and psychological measures

To further examine the impact of amyloid burden on neuropsychiatric outcomes, we performed regression analyses controlling for key demographic variables, including age, sex, education, and marital status. As summarized in Table 2, higher amyloid load was positively associated with subjective memory complaints (MCQ), concerns about AD (CAD), and anxiety (STAI). This suggests that amyloid accumulation, even in the absence of cognitive impairment, may contribute to increased awareness of memory difficulties and heightened anxiety. However, no significant association was found between amyloid burden and depressive symptoms (GDS), further supporting the notion that depression and amyloid pathology may be linked through indirect mechanisms rather than a direct causal relationship.

**Table 2:** Association between amyloid burden and neuropsychiatric outcomes.

	β (95% CI)	SE	P Value
**Model 1: MCQT**			
Composite SUVR	0.765 (0.135-1.395)	0.321	.02
Age	0.045 (0.018-0.072)	0.014	<.001
Female sex	0.353 (0.096-0.610)	0.131	.007
Widowed	-0.140 (-0.569 to 0.289)	0.219	.52
Divorced	0.0004 (-0.354 to 0.355)	0.181	>.99
Single	-0.067 (-0.681 to 0.547)	0.313	.83
Unknown marital status	-0.045 (-0.920 to 0.830)	0.446	.92
Years of education	0.023 (-0.020 to 0.066)	0.022	.30
**Model 2: CAD**	**OR (95% CI)**		
Composite SUVR	6.68 (3.59-12.79)	0.324	<.001
Age	0.92 (0.90 to 0.94)	0.011	<.001
Female sex	1.70 (1.37 to 2.12)	0.112	<.001
Widowed	0.69 (0.49 to 0.97)	0.175	.03
Divorced	0.80 (0.59 to 1.10)	0.157	.16
Single	0.96 (0.57 to 1.71)	0.277	.89
Unknown marital status	0.66 (0.35 to 1.40)	0.353	.24
Years of education	1.00 (0.97 to 1.04)	0.019	.96
**Model 3: GDS**	**β (95% CI)**		
Composite SUVR	0.134 (-0.091 to 0.359)	0.115	.24
Age	0.009 (-0.001 to 0.019)	0.005	.07
Female sex	-0.096 (-0.188 to -0.004)	0.047	.04
Widowed	0.058 (-0.097 to 0.213)	0.079	.46
Divorced	0.190 (0.063-0.317)	0.065	.003
Single	0.369 (0.150-0.588)	0.112	.001
Unknown marital status	0.379 (0.060-0.698)	0.163	.02
Years of education	-0.031 (-0.047 to -0.015)	0.008	<.001
**Model 4: STAI**	**β (95% CI)**		
Composite SUVR	0.719 (0.240-1.198)	0.244	.003
Age	0.001 (-0.019 to 0.021)	0.010	.92
Female sex	0.637 (0.441-0.833)	0.100	<.001
Widowed	-0.096 (-0.423 to 0.231)	0.167	.56
Divorced	-0.277 (-0.547 to -0.007)	0.138	.04
Single	0.174 (-0.291 to 0.639)	0.237	.46
Unknown marital status	0.822 (0.146-1.498)	0.345	.02
Years of education	0.004 (-0.029 to 0.037)	0.017	.81

Abbreviations: CAD, Concerns about Alzheimer's Disease; CI, confidence interval; GDS, Geriatric Depression Scale; MCQT, Memory Complaint Questionnaire Total; STAI, State-Trait Anxiety Inventory; SUVR, standardized uptake value ratio. ᶜ CAD outcome was dichotomized for logistic regression analysis.

To further clarify these relationships, we conducted an additional regression analysis controlling not only for demographic variables but also for subjective memory complaints (MCQ) and concerns about AD (CAD) (Table 3). Even after accounting for these factors, amyloid burden remained significantly associated with subclinical anxiety symptoms (STAI), but not with depressive symptoms (GDS). This finding suggests that the relationship between amyloid accumulation and anxiety is independent of subjective cognitive concerns and concerns about AD, whereas subclinical depressive symptoms appear to be driven primarily by these subjective perceptions rather than amyloid pathology itself.

**Table 3:** Association between amyloid burden and neuropsychiatric outcomes after additional adjustment for subjective stress measures.

	β (95% CI)	SE	P Value
**Model 1: GDS**			
Composite SUVR	0.047 (-0.206 to 0.300)	0.129	.72
Age	0.006 (-0.006 to 0.018)	0.006	.32
Female sex	-0.121 (-0.225 to -0.017)	0.053	.02
Widowed	0.016 (-0.158 to 0.190)	0.089	.86
Divorced	0.269 (0.128-0.410)	0.072	<.001
Single	0.474 (0.231-0.717)	0.124	<.001
Unknown marital status	0.399 (0.056-0.742)	0.175	.02
Years of education	-0.033 (-0.051 to -0.015)	0.009	<.001
MCQT	0.075 (0.063-0.087)	0.006	<.001
CAD (agree vs disagree)	0.156 (0.003-0.309)	0.078	.05
**Model 2: STAI**			
Composite SUVR	0.727 (0.178-1.276)	0.280	.009
Age	0.0001 (-0.023 to 0.023)	0.012	.99
Female sex	0.570 (0.346-0.794)	0.114	<.001
Widowed	-0.238 (-0.614 to 0.138)	0.192	.21
Divorced	-0.166 (-0.472 to 0.140)	0.156	.29
Single	0.359 (-0.168 to 0.886)	0.269	.18
Unknown marital status	0.724 (-0.019 to 1.467)	0.379	.06
Years of education	-0.012 (-0.049 to 0.025)	0.019	.53
MCQT	0.069 (0.044-0.094)	0.013	<.001
CAD (agree vs disagree)	0.488 (0.157-0.819)	0.169	.004

Abbreviations: CAD, Concerns about Alzheimer's Disease; CI, confidence interval; GDS, Geriatric Depression Scale; MCQT, Memory Complaint Questionnaire Total; STAI, State-Trait Anxiety Inventory; SUVR, standardized uptake value ratio.

### 3.5 Mediation analysis of amyloid burden and neuropsychiatric symptoms

To better understand the underlying mechanisms linking amyloid burden with neuropsychiatric outcomes, we conducted mediation analyses to determine whether subjective cognitive complaints (MCQ) and concerns about AD (CAD) played a mediating role in the association between amyloid burden and symptoms of anxiety and depression. These analyses aimed to clarify whether amyloid burden exerts its psychological effects directly or through perceived cognitive concerns.

**Fig. 2:**
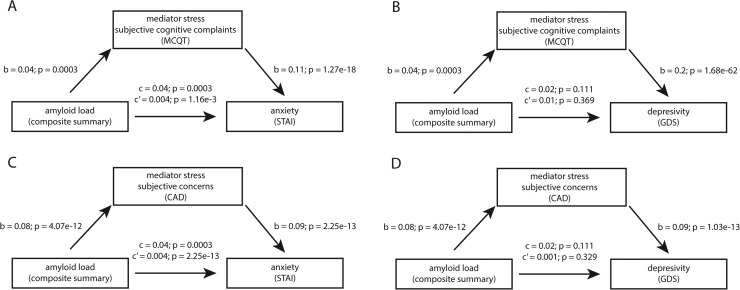
**Mediation Analysis of the Relationship Between Amyloid Burden and Neuropsychiatric Symptoms.** Path diagrams showing direct (c') and indirect effects of amyloid burden on neuropsychiatric outcomes through subjective stress mediators. (A) Amyloid burden shows a significant direct effect on anxiety (STAI) that is not mediated by subjective cognitive complaints (MCQ). (B) Amyloid burden shows no significant direct effect on depression (GDS), but the relationship is partially mediated by subjective cognitive complaints. (C) Amyloid burden shows a significant direct effect on anxiety that is not mediated by concerns about Alzheimer's disease (CAD). (D) Amyloid burden shows no significant direct effect on depression, but the relationship is partially mediated by concerns about Alzheimer's disease. All models adjusted for age, sex, marital status, and education. Path coefficients (b) represent standardized regression weights; c = total effect; c' = direct effect. STAI = State-Trait Anxiety Inventory; GDS = Geriatric Depression Scale; MCQT = Memory Complaint Questionnaire Total; CAD = Concerns about Alzheimer's Disease.

The mediation analysis revealed that anxiety levels, as measured by the STAI, were not significantly mediated by either subjective cognitive complaints (MCQ) or concerns about AD (CAD) (Fig. 2a and c). This indicates that the relationship between amyloid burden and anxiety is direct and not explained by subjective concerns about cognitive decline or AD risk. This finding suggests that amyloid burden has a direct relationship with anxiety symptoms, independent of individuals’ subjective perceptions of their cognitive health or concerns about developing AD. This could indicate a neurobiological effect of amyloid accumulation on anxiety-related processes rather than a purely psychological response to perceived cognitive decline.

Conversely, the mediation analysis showed that subclinical depressive symptoms, as measured by the GDS, were significantly mediated by subjective cognitive complaints (MCQ) and concerns about AD (CAD; Fig. 2, Fig. 2). Although amyloid burden was not directly associated with depressive symptoms, the presence of subjective cognitive concerns and increased worry about AD appeared to contribute to depressive symptomatology. These results suggest that while amyloid burden itself does not directly predict depressive symptoms, individuals who report greater memory concerns or heightened anxiety about AD are more likely to experience depressive symptoms. This highlights the role of perceived cognitive decline in shaping mood-related outcomes, even in the absence of objective cognitive impairment.

## 4 Discussion

This cross-sectional study investigated how elevated β-amyloid (Aβ) burden in cognitively unimpaired older adults relates to neuropsychiatric symptoms - particularly anxiety, depression, and subjective stress regarding Alzheimer’s disease (AD). We found that individuals with elevated amyloid showed slightly higher subjective memory complaints and markedly greater disease-specific worries, aligning with prior work suggesting that preclinical AD pathology can manifest as heightened self-awareness or concern about cognitive decline [22]. Notably, these worries emerged before participants received any formal disclosure of their amyloid status, implying that individuals might sense subtle internal changes linked to Aβ accumulation.

Importantly, the clinical significance of our findings must be interpreted cautiously. Mean STAI scores (approximately 10 out of 24) and GDS scores (approximately 1 out of 15) in both amyloid-positive and negative groups were well below clinical thresholds. The GDS, in particular, showed floor effects that limit our ability to detect meaningful variations in depressive symptoms. Thus, while statistically significant differences emerged, these represent subtle variations within the normal range rather than clinically meaningful anxiety or depression.

The A4 Study's exclusion of individuals with elevated depressive symptoms (GDS > 5) provided a unique opportunity to examine anxiety in a non-depressed sample. Our findings suggest that anxiety symptoms in preclinical AD can be distinguished from depressive symptoms and are not merely manifestations of subsyndromal depression (e.g., psychomotor agitation, irritability). This supports screening for anxiety as a distinct neuropsychiatric domain in older adults, separate from depression screening. However, we note that studies examining populations with greater variability in depressive symptoms have demonstrated associations between depression and AD biomarkers, and our results should not be interpreted as suggesting anxiety is more important than depression in AD risk stratification.

Our findings align with a growing body of literature demonstrating that neuropsychiatric symptoms can precede cognitive decline in AD. Geda and colleagues [18] found that baseline anxiety increased risk for incident MCI in a population-based sample. The Mild Behavioral Impairment (MBI) framework proposed by Ismail et al. [13] similarly positions late-onset neuropsychiatric symptoms as potential harbingers of neurodegenerative disease. Our results extend these observations by demonstrating distinct patterns for anxiety versus depression in their association with preclinical amyloid pathology—anxiety showing a direct statistical association with amyloid burden while depression appears linked only indirectly through subjective cognitive concerns. This differentiation between neuropsychiatric symptom types may have implications for understanding the heterogeneous neurobiological substrates of mood symptoms in preclinical AD.

### 4.1 Neuropsychiatric patterns in Aβ+ vs. Aβ−

While categorical group comparisons did not reveal significant differences in anxiety scores between amyloid-positive and amyloid-negative participants (p = 0.10), regression analyses treating amyloid burden as a continuous variable (SUVR) revealed a significant positive association with anxiety symptoms after controlling for demographic factors (β = 0.719, p = 0.003). This pattern suggests that the relationship between amyloid and anxiety may be more readily detected when preserving the continuous nature of amyloid measurement. In contrast, we found no direct link between amyloid and subclinical depressive symptoms. This pattern is consistent with earlier studies showing minimal group-level differences in psychological outcomes among cognitively normal individuals, even after amyloid-PET disclosure [26,27]. Our findings add nuance by suggesting that subthreshold or domain-specific symptoms (e.g., AD-specific worry) can still manifest when amyloid burden is elevated, supporting the notion that Aβ positivity alone is not necessarily enough to drive a generalized mood disturbance.

### 4.2 Subjective stress as a mediator

One of our key aims was to evaluate whether subjective memory complaints and AD-specific concerns influence the link between amyloid and neuropsychiatric outcomes. Mediation analyses revealed that anxiety remained directly associated with amyloid burden, suggesting potential neurobiological mechanisms - such as network hyperexcitability or neuroinflammation – that may be associated with anxiety symptoms independent of subjective appraisal. Conversely, depressive symptoms appeared indirectly linked to amyloid through subjective stress, in line with research indicating that individuals who perceive cognitive decline or worry about developing AD are more prone to depressive mood states [17].

These observations echo prior findings that subjective stress (e.g., memory complaints, concerns about AD) can magnify the psychological effects of amyloid pathology, even in those without overt cognitive deficits [22,44]. Chronic stress and elevated cortisol, for instance, can exacerbate amyloid deposition [11], while neuroinflammatory and cerebrovascular processes may contribute to anxious and depressive states in the context of AD [45]. Taken together, these data imply a two-track mechanism in early AD: a direct statistical association with subclinical anxiety, and an indirect pathway to depressive symptoms mediated by internal stress and fears about disease progression.

### 4.3 Demographic influences

Beyond amyloid, demographic factors can shape neuropsychiatric risk. Prior analyses indicate that older age is tied to subjective cognitive decline, and some research notes sex differences - with females reporting higher anxiety and memory complaints, whereas males may show more depressive symptoms [22,44,46]. Additionally, social factors like marital status and education can modulate vulnerability to mood disturbances or memory worries [46]. Although our main results did not isolate strong demographic effects, these variables were important covariates in our analyses and should be considered in future longitudinal work.

### 4.4 Clinical and research implications

Our findings underscore the importance of recognizing early psychological indicators in older adults who remain cognitively normal yet have biomarker evidence of AD pathology. Identifying individuals with elevated Aβ who simultaneously exhibit heightened AD-specific fears or anxiety could refine risk stratification and direct supportive interventions - such as counseling or stress-management programs - aimed at mitigating the psychological burden of preclinical AD. Early psychological support may, in turn, lessen depressive trajectories driven by persistent worry or self-monitoring. Moreover, from a research standpoint, capturing both objective biomarkers and subjective stress metrics could enrich predictive models for AD progression. Longitudinal studies are needed to clarify how these subthreshold neuropsychiatric changes evolve, and whether anxiety or disease-specific worries accelerate transitions from unimpaired to mild cognitive impairment or dementia.

Our findings highlight the STAI as a potentially sensitive measure for detecting anxiety symptoms associated with preclinical amyloid pathology. Anxiety assessment instruments vary considerably in their content, with some emphasizing somatic symptoms (e.g., racing heart, shortness of breath) that may be confounded by medical comorbidities common in older adults. The STAI's focus on cognitive-affective components of anxiety (e.g., feeling calm, feeling secure, feeling at ease) may provide a more specific signal in populations where physical symptoms could reflect cardiovascular, pulmonary, or other medical conditions rather than anxiety per se. Future research should directly compare different anxiety measures to determine which instruments are most sensitive and specific for detecting AD-related anxiety symptoms while minimizing confounding from medical comorbidities.

### 4.5 Pathophysiological perspective

Several mechanistic pathways may explain why amyloid correlates more directly with anxiety than with depression at this early stage. Elevated amyloid can disrupt serotonergic and noradrenergic systems crucial for affect regulation [17]. Moreover, microglial activation secondary to amyloid deposition may release pro-inflammatory cytokines linked to anxiety [45]. For depressive symptoms to emerge, however, it appears that subjective stress about memory or the future onset of AD might play a catalyzing role - especially in a sample with relatively low baseline depression risk (GDS ≤ 5). Future studies incorporating tau imaging, cerebrovascular burden, cortisol assays, and genetic risk factors (e.g., APOE ε4 status) could further elucidate the interplay between biological vulnerability and psychological coping.

### 4.6 Strengths and limitations

A key strength of this study is the use of comprehensive psychological assessments, including domain-specific worries about AD, supplemented by robust amyloid-PET data in a well-characterized cohort. However, several limitations temper the generalizability of our findings. First, participants were largely white, educated, and motivated volunteers - limiting the applicability to more diverse populations. Second, while some effects reached high statistical significance, real-world clinical relevance may be modest. Third, a significant limitation is the temporal gap between mood assessment at Visit 1 and amyloid PET imaging at Visit 2, which could have been several weeks apart. Since the GDS captures depressive symptoms from only the past week and the STAI-State measures current anxiety at the moment of assessment, this time lag limits our ability to establish direct associations between amyloid burden and neuropsychiatric symptoms. Mood states may have fluctuated between these assessments due to various psychosocial factors unrelated to amyloid pathology. Fourth, the cross-sectional design precludes causal inferences; we cannot definitively state whether heightened concerns drive subclinical mood changes or if latent neurobiology fosters such worries. Prospective follow-up is essential to determine whether these subclinical symptoms predict faster cognitive decline or progression to mild cognitive impairment.

Our reliance on MMSE scores ≥ 25 to define cognitive normality represents another limitation. Without knowledge of participants' premorbid cognitive baseline, we cannot determine whether an MMSE of 25-30 truly represents preserved cognition or subtle decline from a previously higher level of functioning. This concern is particularly relevant given our highly educated sample (mean education = 16.6 years), where cognitive reserve may mask early neurodegenerative changes. Individuals with high educational attainment may maintain MMSE scores in the 'normal' range despite experiencing meaningful cognitive decline from their personal baseline.

Additionally, we were unable to control for medical comorbidities beyond those captured by the A4 Study exclusion criteria. Given that conditions such as cardiovascular disease, diabetes, and chronic pain are associated with both anxiety and depression in older adults, future studies should incorporate comprehensive comorbidity assessments to better isolate the specific contribution of amyloid pathology to neuropsychiatric symptoms.

Sleep quality, which has bidirectional relationships with both mood symptoms and amyloid accumulation, was not assessed in the available A4 screening data. Future investigations should incorporate sleep measures to disentangle these interrelated factors.

Family history of Alzheimer's disease, which may independently influence concerns about cognitive decline and worry about developing AD, was not included as a covariate in our models. Individuals with affected family members may have heightened vigilance about memory changes regardless of their own amyloid status.

The cross-sectional design precludes causal inferences; our mediation models identify statistical patterns of association but cannot establish temporal precedence or causal directionality. Longitudinal studies are essential to determine whether anxiety precedes, follows, or emerges concurrently with amyloid accumulation.

The A4 Study assessed only state anxiety using a 6-item short form, precluding examination of trait anxiety. Future studies should include both state and trait anxiety measures to determine whether amyloid pathology is more strongly associated with transient versus enduring anxiety characteristics. Distinguishing these could inform whether anxiety in preclinical AD reflects new-onset symptoms versus amplification of pre-existing tendencies.

### 4.7 Future directions

Future studies should incorporate longitudinal designs to determine whether anxiety symptoms and subjective cognitive concerns predict faster progression to cognitive impairment in amyloid-positive individuals. Combining amyloid-PET with tau-PET and functional connectivity analyses could help elucidate the neural mechanisms linking amyloid pathology to specific psychological symptoms. Importantly, intervention studies targeting anxiety and subjective stress in preclinical AD might determine whether addressing these psychological factors can modify disease trajectory.

### 4.8 Conclusion

In conclusion, our results provide evidence that preclinical amyloid pathology may be linked to subtle but meaningful differences in anxiety and disease-specific worry, even in cognitively unimpaired older adults who lack formal knowledge of their amyloid status. Anxiety appears directly related to amyloid burden, while depressive symptoms manifest primarily through the mediation of subjective cognitive concerns and worry about AD. Recognizing and addressing these early psychological markers could foster better emotional support and more effective interventions, ultimately contributing to improved well-being and potentially altering the trajectory of Alzheimer’s disease progression. These findings suggest that the traditional staging of AD with neuropsychiatric symptoms emerging only after cognitive symptoms may need reconsideration. Rather, specific types of neuropsychiatric symptoms - particularly anxiety and subjective concerns - may emerge simultaneously with or even before subtle cognitive inefficiencies become apparent. Recognizing this complex interplay between amyloid pathology, psychological symptoms, and subjective experiences could reshape early detection approaches and intervention strategies for individuals at risk for AD.

## Declaration of the use of generative AI-assisted technologies in scientific writing and in figures, images and artwork

The manuscript was proofread for language, grammar, spelling, and consistency using Claude AI (Claude Opus 4, Anthropic) between March and December 2025. The authors reviewed and edited all AI-assisted content and take full responsibility for the integrity and accuracy of the final manuscript. AI was not used for data analysis, interpretation, or generating scientific content.

## Funding

Dr Vogelgsang received funding from the Alzheimer's Association (AACSF-23-1149842).

## Consent statement

All participants provided informed consent for their participation in the study.

## Data availability statement

Data used in this study were obtained from the Anti-Amyloid Treatment in Asymptomatic Alzheimer's (A4) Study, available through the Laboratory of Neuro Imaging (LONI) Image and Data Archive (IDA) under an approved data-use agreement. Qualified researchers may request access at https://ida.loni.usc.edu/.

## CRediT authorship contribution statement

**Jonathan Vogelgsang:** Writing – original draft, Visualization, Supervision, Methodology, Investigation, Funding acquisition, Data curation, Conceptualization. **Clara Beck:** Writing – original draft, Visualization, Methodology, Formal analysis, Data curation. **Regan Patrick:** Writing – review & editing. **Ipsit Vahia:** Writing – review & editing. **Sara Weisenbach:** Writing – original draft, Supervision, Methodology.

## Declaration of competing interest

The authors declare the following financial interests/personal relationships which may be considered as potential competing interests:

Jonathan Vogelgsang reports financial support was provided by Alzheimer’s Association. Jonathan Vogelgsang reports a relationship with Alzheimer’s Association that includes: funding grants. If there are other authors, they declare that they have no known competing financial interests or personal relationships that could have appeared to influence the work reported in this paper.
